# Performance Analysis of Motion-Sensor Behavior for User Authentication on Smartphones

**DOI:** 10.3390/s16030345

**Published:** 2016-03-09

**Authors:** Chao Shen, Tianwen Yu, Sheng Yuan, Yunpeng Li, Xiaohong Guan

**Affiliations:** School of Electronic and Information Engineering, Xi’an Jiaotong University, 710049 Xi’an, China; twyu@sei.xjtu.edu.cn (T.Y.); neo_yuan@outlook.com (S.Y.); ypli.chn@gmail.com (Y.L.); xhguan@sei.xjtu.edu.cn (X.G.)

**Keywords:** smartphone security, user authentication, behavior analysis, motion sensor, performance evaluation

## Abstract

The growing trend of using smartphones as personal computing platforms to access and store private information has stressed the demand for secure and usable authentication mechanisms. This paper investigates the feasibility and applicability of using motion-sensor behavior data for user authentication on smartphones. For each sample of the passcode, sensory data from motion sensors are analyzed to extract descriptive and intensive features for accurate and fine-grained characterization of users’ passcode-input actions. One-class learning methods are applied to the feature space for performing user authentication. Analyses are conducted using data from 48 participants with 129,621 passcode samples across various operational scenarios and different types of smartphones. Extensive experiments are included to examine the efficacy of the proposed approach, which achieves a false-rejection rate of 6.85% and a false-acceptance rate of 5.01%. Additional experiments on usability with respect to passcode length, sensitivity with respect to training sample size, scalability with respect to number of users, and flexibility with respect to screen size were provided to further explore the effectiveness and practicability. The results suggest that sensory data could provide useful authentication information, and this level of performance approaches sufficiency for two-factor authentication on smartphones. Our dataset is publicly available to facilitate future research.

## 1. Introduction

Smartphone have become omnipresent personal computing platforms for users to access Internet services whenever and wherever. Recent surveys [1,2] show that as more and more private information and security information are stored in smartphones (e.g., 92.8% of Android smartphone users store private information in their smartphones), the risk of information leakage is becoming a major concern for the information society [3]. The most common mechanism to address this problem is authentication. Smartphone users mainly adopt PIN-based passcodes, pattern-based passcodes, or fingerprints as the primary ways, which have been integrated into current smartphone systems. However, most passcodes are simple and easily guessed due to users’ preference for convenience and memorability [4,5]. Some recent studies also showed that users’ passcodes can be inferred through the smartphone onboard sensors [6,7], or through the smudges left on touchscreen surface [8]. The fingerprint-based methods usually require auxiliary hardware, and it is only applied in iOS and a few Android devices. Recently, attackers were able to even get fingerprints from public events with help of a standard camera, and then use these fingerprints for smartphone authentication [9].

Of various potential solutions, a particularly promising technique is the use of sensory data from the smartphone’s onboard sensors, which measures a user’s touch-input action characteristics when inputting passcodes [10,11]. Compared with biometric features for smartphone authentication such as face and touch-interaction behavior (e.g., touch-sliding behavior), onboard sensor behavior does not need special hardware and root access privilege on smartphones to obtain biometric data, and is less sensitive to users’ privacy. Thus it may provide a non-intrusive and implicit solution for enhancing the passcode-based authentication mechanisms.

In this paper, we present a feasibility and applicability study of using motion-sensor behavior for user authentication on smartphones. The rationale behind our work is that touch-input actions from different users would generate different levels of posture and motion change of smartphones which may exhibit the unique behavioral characteristics of individuals. When a user input his/her passcode on smartphones, the sensory data from accelerometer and gyroscope are recorded. We extract descriptive and intensive features for accurate characterization of motion-sensor behavior induced by users’ passcode-input actions, and conduct an empirical feature study to measure the stability and discriminability of these features. We employ three types of one-class classifier to conduct the task of user authentication. We then perform extensive analyses using data from 48 participants with 129,621 passcode-input samples across various operational scenarios and different types of smartphones. We also examine the performance on usability with respect to passcode length, sensitivity with respect to training sample size, scalability with respect to the number of users, and flexibility with respect to smartphone screen size, to further analyze the effectiveness of the proposed approach. It should be noted that we do not propose our motion-sensor-behavior based authentication method as the sole authentication mechanism but rather as a complementary mechanism that can be used to improve security in smartphones. As an example, when a user unlocks his/her smartphone with a passcode, the passcode as well as the sensory characteristics will be examined. The main purpose and contributions of this paper are summarized as follows:

- We present an empirical work analyzing motion-sensor data for smartphone authentication, and analyze the feasibility and applicability of authenticating a user based on the characteristics of motion-sensor data across various operational scenarios and different types of smartphones, which can be easily integrated with existing smartphone authentication mechanisms.
- We model the behavior of accelerometer and gyroscope sensors by proposing descriptive and intensive features, such as descriptive statistics and information entropy of sensor-data sequences, to characterize a user’s passcode-input actions in a robust and accurate manner. These features could lead to a performance boost in stability and discriminability of authentication performance. We also employ three types of one-class classifier to build the authentication model, so that the model can be trained solely on the samples from the legitimate user, and we could examine whether an observed effect is specific to one type of classifier or holds for a range of classifiers.
- We examine the proposed approach in terms of usability with respect to passcode length, sensitivity with respect to training sample size, scalability with respect to the number of users, and flexibility to screen size, to further examine the applicability and generalization capability of the proposed approach.
- A public sensory behavior dataset is established (see Section 4 for availability), not only for this study, but also to foster future research. This dataset contains high-quality behavior data of smartphone sensors from 48 subjects. To our knowledge, this study is the first to publish a shared sensory dataset for smartphone authentication.
- This study systematically evaluates user authentication on smartphones by analyzing behavior of accelerometer and gyroscope sensors, and extensive analyses across various operational scenarios and different types of smartphones show the proposed approach can perform user authentication with a high degree of accuracy. These results suggest that sensory data could provide auxiliary authentication information, and this level of accuracy approaches sufficiency for two-factor authentication for passwords or PINs on smartphones.

The structure of the paper is as follows: Section 2 introduces related work. Section 3 introduces the model. Section 4 describes the data acquisition process. Section 5 introduces the process of data processing and feature extraction. Section 6 details the authentication architecture. Section 7 expounds on the classifier training and testing process, then explains the evaluation methodology. Section 8 presents the experimental results. Section 9 summaries this paper, and discusses future work.

## 2. Background and Related Work

### 2.1. Smartphone Authentication

Currently, the most widely used smartphone authentication mechanisms are PIN-based passcodes, pattern-based passcodes, and fingerprints, which have been integrated into Android or IOS smartphone systems. However, due to the simplicity and easy guessability of the PIN and pattern passcodes [12] (e.g., shoulder surfing attack [13] and smudge attack [8]), and the risk of fingerprint loss from public events (e.g., attackers can get fingerprints from public events with the help of a standard camera [9]), a growing number of biometric features (*i.e.*, signature or gesture based methods [14,15], touch dynamics [16,17], and keystroke dynamics [18,19]) has been applied to strengthen smartphone authentication [14,15,16,17,18,19].

Sun *et al.* [14] developed a two-factor authentication system for multi-touch mobile devices, by asking a user to draw a signature on the touchscreen with multiple fingers to unlock his/her mobile device. The user is then authenticated based on the geometric properties of the drawn curves. A similar idea is presented in [15] to define a set of five-finger gestures for multi-touch device authentication, in which the geometric shape of a given gesture is used and analyzed as the password. They characterized the gesture by classifying movement characteristics of the center of palm and fingertips, and then used classification techniques to recognize unique biometric characteristics of an individual. However, as mentioned in [14,15], these techniques may only be used as weak authentication techniques and are vulnerable to the attackers seeing the users perform their signatures or gestures. In addition, performing multi-finger signatures or gestures on mobile devices with a small display may not be user-friendly.

Bo *et al.* [16] proposed a framework for smartphone authentication based on the dynamics of touch and movement. They extracted features from touch and movement behavior (*i.e.*, pressure, area, duration, position, velocity, and acceleration), and employed a support vector machine (SVM) to perform user authentication task in a lab scenario. Shen *et al.* [17] developed a continuous smartphone authentication system based on users’ touch-sliding operations. The system analyzed four types of touch behavior (*i.e.*, sliding up, down, left, and right), and employed a SVM to learn owner’s touch behavior profile, which was later used for authentication decisions. Although promising results have been shown from these studies in controlled or lab-based scenarios, the reliability and applicability of touch dynamics in real-world scenarios need to be addressed for putting it into more practical settings, and these evaluations pointed out the research of touch behavior analysis are subject to behavioral variability.

Kambourakis *et al.* [18] explored the usage of users’ touch keystrokes for smartphone authentication. They depicted the touch keystroke with the touch speed and distance, and employed the random forest classifier to perform authentication tasks. A subsequent work is presented in [19] to improve the applicability and usability of keystroke biometrics for touch-device authentication. They compared touch-specific features between three different hand postures and evaluation schemes, and showed that the spatial touch features can reduce authentication equal error rates significantly. However, as stated in [18,19], keystroke-based techniques may be vulnerable to attackers familiar with the victim’s typing patterns. In addition, most extant work used data from both legitimate user and impostors for training the classifiers, which may be not suitable for user verification in practice.

### 2.2. Smartphone Authentication through Sensory Data

As sensing and computing capabilities become standard on current smartphones, researchers have begun to collect more types of sensory data on smartphones to build user behavior models and use the model to infer certain contexts, including user authentication [10,11,20]. Table 1 lists some common sensors found in popular smartphones. Smartphone sensors usually include motion sensors, environmental sensors, and position sensors, in which environmental sensors mainly consist of light, temperature, barometer and proximity; position sensors usually include GPS and compass; motion sensors commonly include gravity, accelerometer, gyroscope, and magnetometer. In this evaluation, we focus on motion sensors, which can measure the posture and motion change of smartphones. Some recent studies have shown that the accelerometer can be used to detect coarse-grained motion of a user like how he/she walks for identity authentication [21] and the orientation sensor can be utilized to detect fine-grained motion of a user like how he/she holds a smartphone [22].

In the investigation of smartphone authentication based on the analysis of motion-sensor behavior, there are really two tasks of interest. One task is static authentication, which checks the user only once, typically at unlock or login time. Another is continuous authentication, which checks the user continuously throughout the usage session. The primary focus of previous research has been on the use of motion sensor for identity monitoring [22,23,24], but it is difficult to transfer the work from identity monitoring directly to static authentication, because a rather long observation period is usually required to collect enough sensor data for accurate authentication. To our knowledge, few papers have targeted the analysis of onboard sensor data for static authentication, which will be the central concern of this paper.

Closely related to our purpose are two recent papers by Conti *et al.* [25] and Giuffida *et al.* [26]. Conti *et al.* [25] were the first researchers to use smartphone accelerometer and orientation sensors to authenticate a user when answering or placing a phone call. They characterized smartphone movements, and employed dynamic time warping methods to depict the sensor data. The experiments on 10 subjects showed an impostor-pass rate of 4.5% and a false-acceptance rate of 9.5%. Later on, Giuffida *et al.* [26] presented a framework of combining the traditional keystroke characterization with motion sensors (*i.e.*, accelerometer and gyroscope) to reflect users’ typing behavior, and extracted timing features for authentication. They applied standard classification techniques to perform the authentication task and the experiments on 20 subjects showed that the combination of keystroke characteristics and sensor feature can achieve higher accuracy.

These efforts showed the motion sensor behavior has a rich potential for user authentication on smartphones. In comparison with other biometric features such as face and fingerprint, motion-sensor behavior is less diagnostic. This study, differing from existing work: (1) Aims to provide in-depth analysis of motion-sensor behavior for smartphone authentication in terms of discriminability, stability, and applicability; (2) Examines the authentication performance across various operational scenarios and different types of smartphones; (3) Explores the effectiveness of this technique on usability with respect to passcode length, sensitivity with respect to training sample size, scalability with respect to the number of users, and flexibility with respect to smartphone screen size; (4) Employs one-class classifiers to build the authentication model for making the model be trained solely on the samples from legitimate user; (5) Examines a set of classifiers to compare smartphone authentication performance to explore whether an observed effect is specific to one type of classifier or holds for a range of classifiers; (6) Publishes a shared sensory dataset for smartphone authentication.

## 3. Threat Model

We consider a scenario in which an attacker has physical access to the smartphone, and is already in possession of the passcodes to unlock the device, which means traditional defense has been breached. Thus the smartphone resources (e.g., the applications and private data) are made available to the attacker. For instances, an attacker may steal the application account information and the personal pictures stored in the smartphone. The aim of our work is to develop a sensor-behavior-based smartphone authentication approach by analyzing motion-sensor data in a user’s passcode typing process, without extra involvement and interruption for users. Thus the attacker will be enslaved to a two-factor authentication mechanism when she/he authenticates with traditional passcodes. It should be noted that we mainly consider verifying a user against the smartphone owner, since a smartphone is usually privately owned and may be not shared by others.

## 4. Sensor-Data Acquisition

This section explains how we design passcode-input tasks, set up a data-collection platform, and recruit subjects to perform the tasks. Here we offer details regarding process of data acquisition, because these particulars can best reveal potential biases and confounds to experimental validity [27]. Our dataset is available [28].

### 4.1. Operational Scenarios

To systematically investigate the feasibility of using motion-sensor behavior for smartphone authentication, we design three types of operational scenarios for collecting motion-sensor data, which would roughly cover user’s routine passcode-input actions:

*Hand-hold-input scenario*: Users are asked to operate smartphone for authentication when sitting or standing steadily.
*Table-hold-input scenario*: Smartphones are placed on the desktop, and users were asked to perform authentication actions using a single hand.
*Hand-hold-walk scenario*: Users operate smartphones and perform passcode-input action for authentication when walking.

### 4.2. Apparatus

We establish a free experimental environment on Android smartphones and develop an App as uniform hardware and software platform for the collection of motion-sensor data. We set up three different types of smartphones, each operating on Android 4.4.x. The smartphones are a Huawei Mate7 with a 6.1-inch screen, 1.5 GHz processor, and 2 GB RAM; a Samsung N7100 with a 5.5-inch screen, 1.6 GHz processor, and 2 GB of RAM; and a HongMi 1s with a 4.7-inch screen, 1.6 GHz processor, and 1 GB of RAM.

The smartphone App, written in Java, prompts a user to conduct an authentication (*i.e.*, smartphone unlocking) task. During data collection, the application displays the task in a full-screen window on the smartphone, and records (1) the touch-input actions; (2) the motion-sensor data (*i.e.*, accelerometer and gyroscope) during touch-input actions; and (3) the timestamps of the operations. The default timing clock is used to timestamp touch-input actions [29], which has a resolution of 10 ms, corresponding to 100 updates per second.

### 4.3. Stimulus Materials

Designing passcode-input tasks for such an evaluation is subtle. It is often more realistic to let subjects choose their own passcode inputs. Yet data collection becomes harder since different impostor samples would be needed for every task. Some studies suggest that letting subjects choose their own tasks makes it easier to distinguish them [30,31]. If this is true, then letting subjects choose their own tasks can bias the results of an experiment designed to assess performance on an arbitrary task. Thus we decide the same tasks would be undertaken by all subjects.

To make passcode-input tasks representative of typical and diverse combination of input actions, our stimulus materials consist of three passcodes with different lengths, 0-4-3-9, 0-1-4-7-8, and 0-1-4-3-6-7, and require subjects to type without hyphens to unlock the smartphone. The same passcodes are assigned to all subjects, as opposed to having each subject select passcodes of their own. The reasons for this choice are that: (1) Self-chosen passcodes may be of different lengths, which make users’ typing hard to compare; (2) Self-chosen passcodes may be easy to type (or particularly hard to type), which may introduce biases that are difficult to control; (3) Same passcode can make each subject be treated as impostor for other subjects, putting the authentication test on an equal basis; (4) Same passcode can reduce the effect of experimental control over unanticipated biases.

It is worthy of note that our passcodes are not unique. However, the passcode sequences span the keypad, and a finger typing this sequence travels both of the diagonals, and a vertical. Besides, since we had only one chance to accomplish every passcode, we chose a passcode from which we can learn most. The passcodes are chosen to cause smartphone users to perform a wide variety of finger movements on touchscreen.

### 4.4. Subjects

We recruited 48 volunteers (29 males and 19 females) from among students and faculty from our university. All subjects were skilled smartphone users with at least one year’s experience, and five of them were left-handed. The age ranged from 18 to 50 years (mean = 25.6, s.d. = 4.2).

### 4.5. Data Collection Process

Subjects are required to conduct fifty-four rounds of data collection (three operational scenarios × three smartphones × two rounds for each subject × three types of passcodes), and to wait at least one day between two rounds (ensuring that some day-to-day variation existed within our data). In each round of data collection, subjects are asked to type the passcode about 50 times on the number pad (under the application of smartphone unlocking). All 48 subjects remained in the study, and each subject contributed around 2,700 passcode-input samples (mean = 2721, median = 2758, min = 2601, max = 2822, and s.d. = 33.7). Subjects took between 90 and 120 days to complete the data collection, and the final dataset contains 129,621 authentication samples from 48 subjects. During data collection, when subjects input a passcode on the touchscreen, for each number of the passcode, the application records the tapped number, the timestamps of action starting (ACTION-DOWN) and action ending (ACTION-UP) (*i.e.*, the down and up of a passcode-input action), and the data sequences of motion sensors during the period of ACTION-DOWN and ACTION-UP for the passcode-input action. For each motion sensor, three data sequences are collected to represent the data from three axes of the sensor.

Subjects are told that if they needed a break or needed to stretch their fingers, they were to do so after they have completed a task. This is intended to prevent artificially inconsistent passcode-input actions in the middle of a task. Subjects are admonished to focus on the task, as if they are unlocking their own smartphones, and to avoid distractions while the task is in progress. Besides, in the table-hold-input scenario, we observe that all subjects tap with her/his dominant hand. While in other two scenarios, we find that all the subjects hold the devices with one hand and type with the other hand, and the smartphone is in the portrait mode. In addition, to trigger the interaction in a natural way, the subjects are explained the purpose of the study after finishing all data collection.

## 5. Motion-Sensor Behavior Analysis

### 5.1. Preprocessing

#### 5.1.1. Gravity Filtering

Generally, raw motion-sensor data include the gravity component, which may make the obtained sensor data hard to accurately reflect the posture and motion change of smartphones. Since researchers usually consider gravity component as a constant component and take motion data as alternating components [32], the filtering technique is employed to remove the gravity components. Here we employ the Kalman filter method, which is a recursive way of estimating optimal value of state variables, to obtain an unbiased estimated value of motion-sensor data. Specifically, the gravitational component embedded in raw sensor data can be reduced in each of three axes (*X*, *Y*, *Z*) of the sensors (*i.e.*, accelerometer and gyroscope sensors) by following steps:

*Step 1*: We compute predicted value of sensor data *P* at time *t* by using estimated value at time *t*−1:
  (1) P(t|t−1)=A⋅P(t−1|t−1)+B⋅U(t)
  where *P*(*t*|*t*−1)={*X*(*t*|*t*−1), *Y*(*t*|*t*−1), *Z*(*t*|*t*−1)} is the predicted value of sensor data at three smartphone axes, *U*(*t*) is the control value at time *t*, and *A*, *B* are the coefficient matrices.
*Step 2*: We calculate deviation *D* of the predicted value at time *t*:
  (2) D(t|t−1)=A⋅D(t−1|t−1)⋅A′+Q
  where *A’* is the transposed matrix of *A*, Q is the process deviation.
*Step 3*: We obtain unbiased estimated value of sensor data *P*(*t|t*) at time *t* based on the measured value *Z*(*t*) and the predicted value *P*(*t*|*t*−1), and also update the deviation of *P*(*t|t*):
  (3) $$\begin{array}{l} P(t|t)=P(t|t-1)+Kg(t)\cdot (Z(t)-H\cdot P(t|t-1))\\ Kg(t)=\frac{D(t|t-1)\cdot H\prime }{\left(H\cdot D(t|t-1)\cdot H\prime +R\right)}\\ D(t|t)=(I-Kg(t)\cdot H)\cdot D(t|t-1) \end{array}$$
  where *Kg* is the Kalman Gain coefficient, *R* is the deviation of the measurement, and *H* is the system matrix.

#### 5.1.2. Wavelet Denoising

In addition, sensor signals usually inevitably contain non-stationary noise which makes the signals exhibit multiple peaks. This would directly lower the accuracy of feature modeling and user authentication. Thus we apply a wavelet-based denoising method to mitigate the signal mutation instead of traditional Fourier analysis method, since the later one converts a signal in the frequency domain at a certain time point, but the mutation and noise usually affect the entire spectrum of the signal. Consider how the wavelet-based denoising method is applied to mitigate the effect of non-stationary noise in sensor signals:

*Step 1*: We select suitable wavelet functions to decompose the signals into *N* levels and extract low-frequency coefficients of every level and high-frequency coefficient of the *N*th level.
*Step 2*: We employ the threshold analysis to filter decomposed signals.
*Step 3*: We use an inverse wavelet transform on the filtered-decomposed signals to reconstruct the original signal.

### 5.2. Sensor-Behavior Features

The sensor data cannot be used directly by a classifier for user authentication. Instead, behavior features are extracted from these data. For each passcode-input action, we extract motion-sensor behavior data and depict them by two feature sets: descriptive features and intensive features. Descriptive features characterize the motion patterns of passcode-input actions with meaningful statistics. For instance, the range of a *y*-axis gravity series indicates the angle of smartphone rotation around *y*-axis, which can differentiate users with different rotation angles around that axis. Intensive features depict the intensity and complexity of passcode-input actions. For example, the energy of a sensor-data sequence is calculated by summing up the squared magnitudes of FFT (Fast Fourier Transform) components, which is a metric of action intensity; the entropy of a sensor-data sequence is calculated with Shannon entropy, which measures the complexity of a passcode-input action. Some of these features are different from the conventional features, such as energy and entropy of a sensor-data sequence, which can characterize different passcode-input actions in an accurate and robust manner. For each input action of a passcode, there are a total of 96 features (6 data sequences × 16 features for each data sequence), and these features are taken together to form a feature vector to characterize the input action. Table 2 summarizes the extracted features from each data sequence in this study (with feature dimensionality in parentheses).

### 5.3. Empirical Feature Study

The underlying assumption that motion-sensor behavior can be used as a source for authentication is that the change of motion-sensor data by users’ passcode-input actions should have strong dependency on their identities, which also means sensor features can be used to discriminate among different users. Thus we compute a metric of informativeness [33] for each of sensor-behavior features to examine how users’ identities differ with respect to t features. We define this metric as the relative mutual information between the feature *f_i_* and a user’s identity *D_j_*:

(4) $$I_{f_{i},D_{j}}=\frac{I\left(f_{i};D_{j}\right)}{H\left(D_{j}\right)}=\frac{H\left(D_{j}\right)-H\left(D_{j}|f_{i}\right)}{H\left(D_{j}\right)}=1-\frac{H\left(D_{j}|f_{i}\right)}{H\left(D_{j}\right)}$$

where $I\left(f_{i};D_{j}\right)$ refers to the mutual information between feature *f_i_* and the identity *D_j_*, $H\left(f_{i}\right)$ and $H\left(D_{j}\right)$ are the entropies of the feature and the identity, respectively. For each feature, this metric is calculated as a value between 0 and 1, where 0 means that the feature carries no identity information, and increases as the correlation between the feature and the identity becomes stronger, in which 1 means the feature determines the identity.

To calculate $I\left(f_{i};D_{j}\right)$, we first convert the feature to discrete variables. Specifically, we use 100 equally spaced bins to span the feature with the range from the 10% quantiles to 90% quantiles. Then we compute the mutual information between the feature and the identity using the Equation (4). The above process is then repeated for all pairs of the features and users’ identities. Here the mutual information are computed over 43,200 passcode-input samples from 48 subjects, and we set a threshold as 0.5, in which case the features with $I\left(f_{i};D_{j}\right)$ > 0.5 were selected.

The analysis results of sensor-behavior features with respect to users’ identities are presented in Table 3. Due to space limitations, here we present the 20 top-performing features, which are rank-ordered from best to worst. We can observe that the sensor-behavior features appear to be good metrics since their relative mutual information is much larger than 0.5. This means that these features are good in distinguishing data samples from different users. In addition, the most informative features are energy and entropy of acceleration data in *y* and *z* coordinates. The energy features provide more informativeness than the entropy features, which is probably due to the fact that the energy features measure the intensity of a passcode-input action. We also find that some descriptive statistics of sensor-data sequences, like mean, minimum, maximum, variance, and range, exhibit a certain degree of discriminability, but kurtosis, skewness and cross-mean rate are not as qualified. This may be due to the reason that these features are sensitive to the position of passcode-input actions, and the sensor-data sequence of different passcode-input actions may have differentiable mean values, but generate similar variances (e.g., button ‘4’ and ‘6’), thus may make it hard to distinguish among users.

It should be noted that these rankings do not mean that few top-ranked sensor-behavior features constitute the most informative collection of the features. It is mostly considered that one can gain more information by combining the features that complement each other. Here we select the features with the relative mutual information larger than 0.5 for our authentication task.

## 6. Authentication Architecture

**Figure 1:**
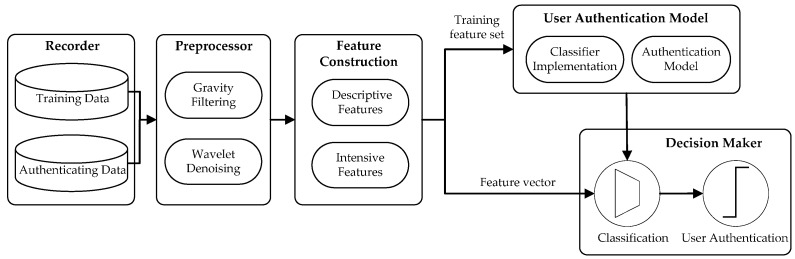
System architecture of our sensor-based smartphone authentication approach.

As shown in Figure 1, our sensor-based smartphone authentication approach mainly consists of five modules-data recorder, data preprocessor, user authentication model, and decision maker. The design of the first three components is straightforward. The main task of the recorder is to record users’ raw motion-sensor data when inputting passcodes on smartphones, while the preprocessor module obtains the stable and clean sensor information and feature-construction module extracts behavioral features. The focus of this section is on the design of the classifier, user authentication model, and that of the decision maker.

### 6.1. Classifier Implementation

Consider the scenario in Section 3, the proposed authentication approach refer to a two-class classification problem (legitimate subject *vs*. impostors), but usually only data from the legitimate subject (*i.e.*, owner of a smartphone) are available for training the authentication model, and the data from impostors is very limited. Therefore, a more appropriate solution in practice is to train the authentication model on the data only from the legitimate user, and then use this model to detect impostors. Thus here we considered the authentication task as a one-class classification or novelty detection problem [34].

#### 6.1.1. Classifier 1: One-Class Support Vector Machine

A one-class Support Vector Machine (SVM) classifier uses a kernel function to map data into a high dimensional space, and considers the origin as only sample from other classes. In the training stage, the classifier is established by using the training vectors with the RBF kernel function, and the SVM parameter and kernel parameter are set to 0.06 and 0.02, respectively. In the testing stage, the classifier projects the test vector onto the same high-dimensional space, and computes the distance between the test vector and the linear separator as the classification score.

#### 6.1.2. Classifier 2: Neural Network

A single hidden layer neural network is used in this evaluation. In the training phase, a network is built with p input nodes, one output node, and (2p + 1) hidden nodes. The network weights are randomly initialized between 0 and 1. The classifier is trained to produce ±1.0 on the output node for training feature samples. We train the classifier for 1000 epochs using a learning rate of 0.001. In the testing phase, the test sample is run through the network, and the output of the network is recorded as the classification score.

#### 6.1.3. Classifier 3: Nearest Neighbor

A nearest-neighbor classifier models a user’s motion-sensor behavior based on the assumption that the new feature samples from the user will resemble one or more of those in the training data. In the training phase, the classifier estimates the covariance matrix of training feature samples, and the nearest-neighbor parameter k is set as 3 after comparative studies. In the testing phase, the classifier calculates Mahalanobis distances, and the average distance from the new sample to the nearest samples is used as the classification score.

### 6.2. Authentication Model

The motion-sensor data is collected for the legitimate user whose behavior we are trying to model. Here we build the authentication model for each type of passcode as follows:

*Step 1*: For a certain type of passcode, we extract sensor-behavior features of every tapped action in that passcode from the training data.
*Step 2*: We combine the sensor-behavior features together to form a feature vector to represent each passcode sample.
*Step 3*: We take these vectors as the training set to train our classifier, to obtain the authentication model for this type of passcode.

### 6.3. Decision Maker

After building the authentication model, we apply this model to detect whether the current user’s sensor behavior is normal or anomalous. During the authentication process, a legitimate user’s behavior profile, generated from the sensor data in the training phase, is compared against current user’s behavior. If there is a significant difference between these two profiles, the current behavior is considered as an anomalous one. Specifically, we first take the feature vector of a testing passcode sample to the authentication model for acquiring the classification score. Then a threshold is set to determine the authentication decision: the classification score over the threshold indicates an impostor, while the classification score under the threshold indicates a true user.

## 7. Evaluation Methodology

### 7.1. Training and Testing Procedure

Considering the scenario as mentioned in Section 6.1, we started by designating one of our 48 subjects as the legitimate user, and the rest as impostors. We train and test each classifier as follows:

*Step 1*: We train the classifier on a randomly-selected half of the feature samples from the legitimate user, to build a profile of the legitimate user*.*
*Step 2*: We test the ability of the classifier to recognize the legitimate user by calculating classification scores on the remaining half of feature samples from the user. We record these scores assigned to each sample as user scores.
*Step 3*: We test the ability of the classifier to recognize the impostors by calculating classification scores on the feature samples from all impostors. We record these scores assigned to each sample as impostor scores.

This process is then repeated, designating each of other subjects as the legitimate user in turn. Since we use a random sampling to divide the data into training and testing sets, we repeat the above procedure twenty times to account for the effect of this randomness, each time with an independent draw from the entire dataset.

### 7.2. Calculating Classifier Performance

To convert these classification scores into aggregate measures of classifier performance, we calculate the false-acceptance rate (FAR) and false-rejection rate (FRR). In our evaluation, the FAR is computed as the ratio between the number of false acceptances and the number of test samples from impostors; the FRR is computed as the ratio between the number of false rejections and the number of test samples from legitimate users. We also brought FAR and FRR together to generate a graphical summary of performance known as ROC curve [35].

Whether or not a feature sample produces an alarm depends on how the threshold on classification score is chosen. A classification score over the threshold indicates a legitimate user, while a score under the threshold indicates an impostor. In our evaluation, threshold is set to be a variable ranging from [−1, 1] to obtain the ROC curve. We choose the threshold to be the default value of 0.0 to calculate the FAR and FRR, and we also compute equal-error rate (EER) at the sensitivity of the classifier where FAR equals FRR.

## 8. Experiments and Analysis

This section presents an objective evaluation on the effectiveness of the proposed approach, in terms of accuracy with various classifiers and across various operational scenarios, usability with respect to passcode length, sensitivity with respect to training sample size, scalability with respect to the number of users, and flexibility with respect to smartphone screen size.

### 8.1. Smartphone Authentication across Various Operational Scenarios

#### 8.1.1. Method

In this evaluation, we conduct a user authentication experiment to evaluate our proposed approach across three different operational scenarios (as discussed in Section 4.1). In each scenario, we have about 43,200 passcode samples from 48 subjects, and use a 5-digit passcode and Samsung N7100 as the test bed. Besides, we use the evaluation methodology in Section 7 to perform the authentication experiment, and apply each of three classifiers in three evaluations, with the inputs respectively set to be the feature spaces obtained from three operational scenarios.

#### 8.1.2. Results and Analysis

**Figure 2:**
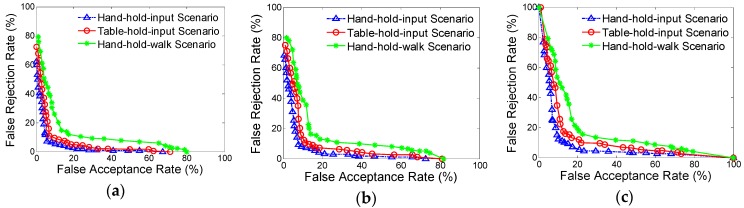
ROC curves for three different operational scenarios by using three types of classifiers: (**a**) support vector machine, (**b**) neural network, and (**c**) k-nearest neighbor.

Figure 2 and Table 4 show the ROC curves and average FARs and FRRs of the authentication task across different operational scenarios, with standard deviations in parentheses. Each panel displays the authentication results by using each of three classifiers. The best authentication error rates (in terms of both FARs and FRRs) in every operational scenario are less than 12%, which indicate that there do exists informative information in motion-sensor behavior for smartphone authentication. The hand-hold-input scenario has the best performance among the three scenarios, and the performance in table-hold-input scenario is relatively better than that in hand-hold-walk scenarios. Specifically, the best performance in hand-hold-input scenario has a FAR of 5.01% and a FRR of 6.85%, obtained by the one-class SVM classifier. This result is very promising, and also is competitive with the best results previously reported [25,26]. With incremental improvements and investigation on its security and usability (e.g., outlier handling), it seems possible that motion-sensor behavior could be used as, at least, a source for an auxiliary authentication technique, such as an enhancement for conventional pin-based or pattern-based authentication mechanisms. Besides, in table-hold-input scenario, since the smartphone is placed horizontally on the desktop and the support force of desktop is uniformly distributed over the back of the screen, the smartphone would generate a smaller posture change than that in hand-hold-input scenario when inputting the passcode, which may lead to a decline of authentication accuracy. While in hand-hold-walk scenario, the sensor data may get affected by people moving, which would introduce additional noise into sensor data. This may be the reason why the authentication error rates in this scenario are worse than those in other two scenarios.

The one-class SVM classifier has a better performance (on both FAR, FRR and standard deviation) than all other classifiers. One-class SVM can well capture the density and modality of a hypersphere of normal behavior, and its kernel functions and support vectors enable it to detect outliers in the case where the data is not linearly separable and to find informative features with a small training set [36]. While the worst is by nearest neighbor classifier, this could be due to the lack of self-learning ability compared with other methods in this study.

#### 8.1.3. System Implementation and Overhead Analysis

We successfully implement an application based on our approach into a smartphone operating system. The smartphone (testbed) is a Samsung N7100 with 5.5-inch screen, 1.6-GHz quad-core processor, and 2 GB of RAM, which runs an Android 5.0 operating system. The application replaces the PIN-based unlocking application, and runs as a two-factor authentication mechanism. Whenever the user types a passcode for unlocking the smartphone, the application monitors and records (1) the passcode-input operations; (2) the motion-sensor data (*i.e.*, accelerometer and gyroscope) during the passcode-input actions; and (3) the timestamps of the actions. Then the application first checks the typed passcode-string, and if it matches, the application computes the authentication score based on the sensor-behavior features. Kernel APIs’ calls are then utilized to authorize or reject the smartphone unlocking accordingly.

Besides, we analyze the system performance and overhead of our implementation in terms of CPU cost, memory cost, and battery usage. We first record the computational cost of CPU in the modules of data preprocessor, feature construction, authentication model, and decision maker. For each legitimate user in the training stage, the data preprocessor and feature construction modules on 50 passcode samples (with six digits) take about 3.82 s, and the authentication model takes only 5.12 s to build the model. Then in the testing stage, the first two modules on a passcode (with 6 digits) take about 301 ms, and the decision maker module takes about 112 ms to make the authentication decision. We then calculate the memory cost by using an Android debugging tool Adb [37]. We find the application consumes about 2 MB of memory resource, and an average of about 0.07 KB is consumed for every testing sample. Besides, another issue to concern is the space for storing user profiles. In our system, sensor feature template of a user consumes 0.19 KB, which is relatively light on a smartphone. We also monitor the battery usage by using the embedded Android APIs (*i.e.*, PowerManagerService) on 20 users for 7 days each. The average per-day battery consumption of the application is less than 1.7% which is relatively negligible as compared to other everyday-used applications (e.g., the web browser which consumes about 30% of battery in our observation). Thus we can observe that the system overheads of our approach are minor.

### 8.2. Usability to Passcode Length

#### 8.2.1. Method

Passcode length in smartphone authentication corresponds to the number of input actions required to form a data sample. The length plays an important role in smartphone authentication since it represents the tradeoff between security (authentication accuracy) and usability (authentication time). The shorter passcode length indicates better user acceptability; however, short passcode length usually means small amounts of sensor data for authentication, which may lead to low authentication accuracy. To examine the effect of passcode length on authentication performance and to analyze the usability, we used three datasets with different passcode lengths (which are set to be 4, 5, and 6 as discussed in Section 4.3), and trained and tested the authentication model in hand-hold-input scenario using one-class SVM classifier. Besides, we used the Samsung N7100 as the test bed, and the evaluation methodology is same as the one discussed in Section 7. In this way, we considered the authentication performance as a function of passcode length, so that authentication performance at different passcode length can be evaluated and compared.

#### 8.2.2. Results and Analysis

**Figure 3:**
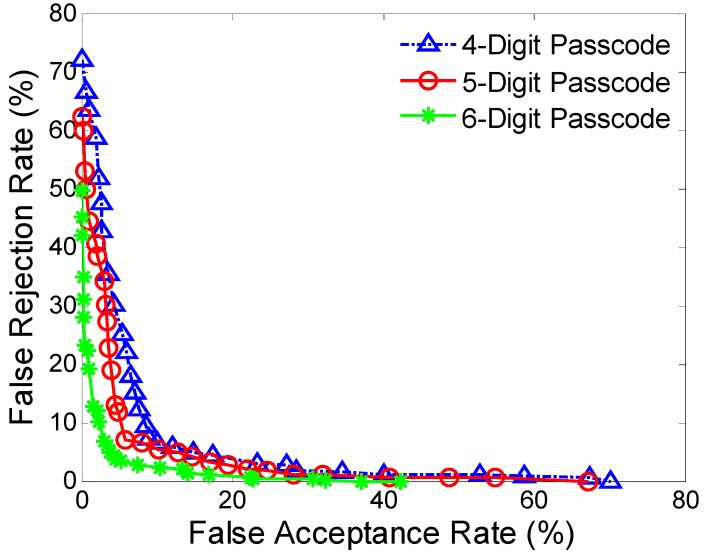
ROC curves at different passcode lengths.

Figure 3 and Table 5 show the ROC curves and average FARs and FRRs at different passcode lengths. The table also includes average authentication time which corresponds to the passcode length.

The results show a trend that the authentication accuracies become better as passcode length increases. The FAR and FRR obtained at the passcode length of 4 is 8.69% and 9.47%, with an average authentication time around 1.52 s. As the passcode length increases to 6, the FAR and FRR reduce to 3.92% and 4.97%. Although long passcode length could result in high authentication accuracy, correspondingky requires a long authentication time, which may affect the usability. Thus a tradeoff should be made between the accuracy and the time required to make the authentication decision, especially in actual deployment scenarios. Besides, login times of typical passwords for authentication on computers are usually well below 10 s [38]. We note that, in our study, the authentication time of 2.89 s (corresponding to a passcode length of five digits) may be acceptable for smartphone authentication, and the FAR of 5.01% and FRR of 6.85% appears to be sufficient for practical application, especially considering sensor behavior as a second factor for smartphone authentication. In addition, the difference between the standard deviations of FRRs and FARs for two passcode lengths becomes smaller when passcode length increases, which indicates that with an increase in passcode length, authentication accuracy becomes more robust and stable.

### 8.3. Sensitivity to Training Sample Size

#### 8.3.1. Method

Training sample size refers to the number of data samples in the training set for building the authentication model. If training sample size is too large, the training process will be unrealistically long and computationally expensive. For another, if training samples are not sufficient, there will have a significant difference between training error and generalization error. To investigate the effect of training sample size on authentication performance, we set the training sample size to 10, 20, 30, 50, and 70, and employ one-class SVM classifier to conduct authentication tasks in hand-hold-input scenario. We use the Samsung N7100 and a 5-digit passcode as the test bed, and the evaluation methodology is same to the one in Section 7.

#### 8.3.2. Results and Analysis

**Figure 4:**
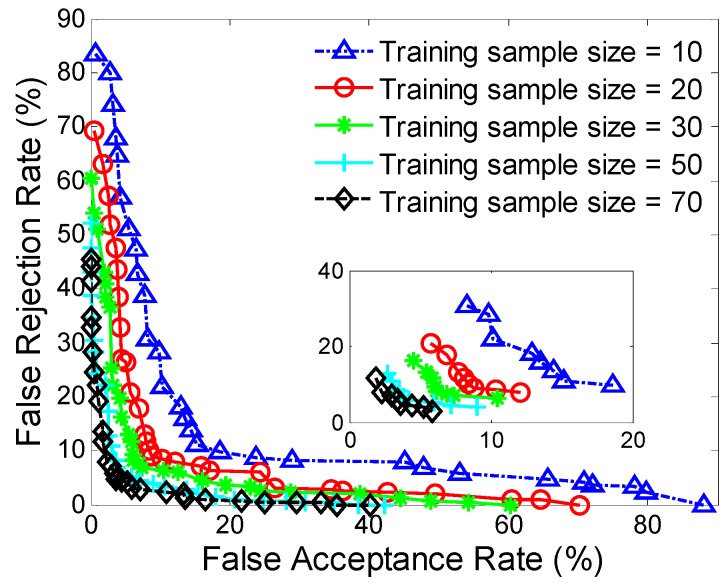
ROC curves against different training data sizes.

Figure 4 and Table 6 show ROC curves and average FARs and FRRs against variable training sample size, to illustrate the sensitivity of the proposed approach to training sample size.

It is as expected that the authentication accuracy for small training sample size is poor, but improves as training sample size increases. Specifically, with 20 samples in the training set, the FAR and FRR are high, up to 9.02% and 10.33%; while with 50 samples in the training set, the FAR and FRR are reduced by about 50%, down to 5.01% and 6.85%. Two conclusions may be drawn from the above observations: (1) Training sample size is important and does have an effect on authentication accuracy; (2) There is a steady increase of authentication performance as training sample size increases.

It can also be seen that if more than 50 samples are used for training, little performance improvement is gained by increasing training sample size, and the accuracy gradually becomes saturated. This result shows that authentication accuracy may be close to optimum if about 50 training samples are used. However, other related work showed that this may be not the case if domain-specific information is exploited in the learning and feature extraction process [39]. It should be noted that a large training dataset usually means a long training process, which may limit its applicability in practice. Thus a balance should be struck between authentication performance and applicability for this technique.

### 8.4. Scalability to User Space

#### 8.4.1. Method

Scalability of user space in sensor-based smartphone authentication refers to the ability of sensor-behavior features being enlarged to accommodate a growth of user space. It is usually true that with an increase in size of user space, there is a higher chance that two users will have similar profiles. Thus one may wonder how the space of profiles fills in as the number of users increases, and further investigate the scalability of our approach to the size of user space. To achieve this goal, we set the size of user space to be a variable ranging from 2 to 48, and employ one-class SVM classifier to conduct the authentication experiment using the evaluation methodology in Section 7. Specifically, we use the Samsung N7100 and 5-digit passcode in the hand-hold-input scenario as the test bed, and for each such number, we repeat the evaluation twenty times with a randomly selected collection of users.

#### 8.4.2. Results and Analysis

**Figure 5:**
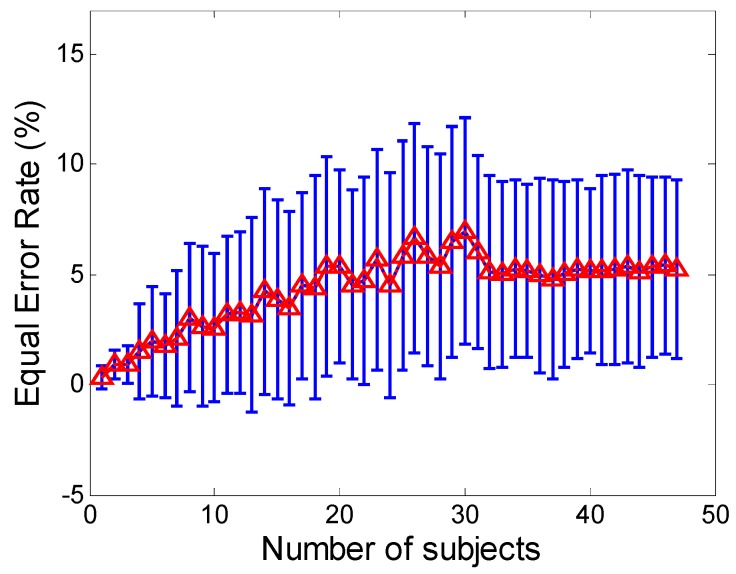
ROC curves against different training data sizes.

The EERs with different sizes of user space, together with their 95% confidence intervals, are presented in Figure 5. The results show the authentication error rates increase as the size of the user space becomes larger, especially for small user space sizes.

Specifically, there is a significant increase in the authentication error rate in the interval between two users and 25 users. This is as expected, since the larger the number of legitimate users usually means the higher the probability that two legitimate users have similar profiles. Also, we observe that when the user size is larger than about 31 users, the authentication error rates become relatively stable, and only small fluctuations with the error range are apparent. These results indicate that the user size in our analysis should be (at least) larger than 31, in which case the influence of user space may be minimal. These results also indicate that our subject size is located in a range where the influence could be negligible. The confidence interval is computed as the mean plus-or-minus 1.96 standard errors. The standard error of the mean is estimated as ($\sigma /\sqrt{n}$), where σ is the standard deviation of the EERs of 48 subjects, and n is 48. The 1.96 multiplier is the quantile of the normal distribution corresponding to a 95% interval [40]. Since the confidence interval becomes gradually stable as the user space exceeds 31, this further demonstrates the authentication performance become relatively stable, and our approach is robust to the increasing user space.

### 8.5. Flexibility to Smartphone Screen Size

#### 8.5.1. Method

Authentication accuracy may be influenced by different sizes of smartphone screen. Intuitively, the area of an input action with a smaller screen size is expected to decreasing the uniqueness of the input action, which may affect authentication accuracy. Here we use three smartphones with different screen sizes: a Huawei Mate7 with a 6.1-inch screen, a Samsung N7100 with a 5.5-inch screen, and a HongMi 1s with a 4.7-inch screen. We then employed one-class SVM classifier to conduct the user authentication task across these smartphones in a hand-hold-input scenario. We use the 5-digit passcode for training and testing the authentication model, and the evaluation methodology is similar to the one in Section 7.

#### 8.5.2. Results and Analysis

**Figure 6:**
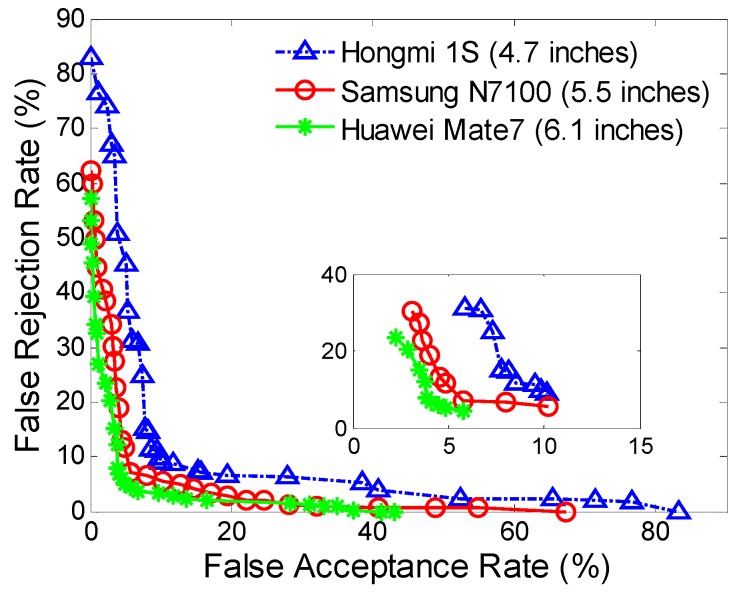
ROC curves at different smartphone screen sizes.

Figure 6 and Table 7 show ROC curves and average FARs and FRRs against three different smartphone screen sizes.

The smartphones with larger screen sizes (Samsung N7100 and Huawei Mate 7) have much better performance than the smartphone with a smaller screen size (HongMi 1S). This is probably due to the reason that input actions on a larger area have better uniqueness and appear more robust than the ones with smaller areas. Specifically, the FAR and FRR with the screen size of 4.7 inches (*i.e.*, HongMi 1S) are 9.74% and 11.09%; while the screen size enlarges to 6.1 inches (*i.e.*, Huawei Mate7), the FAR and FRR reduce to 4.53% and 5.89%, and the corresponding ROC curve is obviously lower.

The figure also reveals that at some points, the performance of Samsung N7100 is a little superior to that of the Huawei Mate7 (e.g., the red-circle line is lower than the green-star line at some points in the subfigure). The cause of this issue is not obvious; it could be an artifact of our smartphones’ motion characteristics and would disappear with different or more smartphone screen sizes. Besides, we try to take smartphone screen size as the variable of interest in this evaluation, but we note that other factors (e.g., sensor types and qualities across different smartphones) might obscure or distort our results. It is notable that the results here only provide preliminary comparative results and should not be concluded that the performance of our proposed approach on larger screen sizes is always better than that on smaller ones. Further comparisons and analyses on both controlled and realistic datasets would be necessary to determine whether such factors actually do confound the experiments, but the existence of these factors does suggest that the effects of the factors like screen size are not always easy to predict.

## 9. Discussion and Conclusions

This work is the first study of evaluating and investigating motion-sensor data for smartphone authentication across various operational scenarios and different types of smartphones. Extensive experiments examine the reliability and practicability of the proposed approach, and show that the approach can achieve a FAR of 3.92% and FRR of 4.97% in some cases. However, this result is still less than the European standard for commercial biometric technology (which requiring a FAR of 0.001% and a FRR of 1% [41]). Thus, further progress is needed before we can depend solely on motion-sensor behavior as a standalone authentication mechanism on smartphones. But these findings do suggest that sensory data could provide useful authentication information, and this level of accuracy approaches sufficiency for two-factor authentication for passwords or PIN numbers on smartphones.

We analyze the effect of passcode length on authentication performance. The authentication accuracies become better as passcode length increases, and the reduced standard deviations indicate better robustness. However, we note that the time needed for completing an authentication increase with the increase of passcode length, which means a balance need to be made between authentication accuracy and authentication time. One possible way of improving this problem is to employ some newly developed tactics from “streaming classification” algorithms [42,43], by which we may be able to use less data to make authentication decisions with acceptable levels of accuracy.

We examine the effect of training data size on smartphone authentication performance. The authentication accuracies improve as the training data size increases. However, a large training sample size will usually result in long training process for users and relatively-high computational complexity. In our evaluation, the FAR and FRR are less than about 9% when using 30 samples for model training, which indicates this technique supports the usage of a relatively small set of training data to authenticate users. One possible way to further decrease training data size while keeping accuracy may be to reduce the noise in raw data for obtaining higher quality data, which might build an accurate authentication model over smaller amounts of training data.

Another important issue about our approach concerns its scalability. We explore the authentication accuracy against different sizes of user space. The EER increases as the user space becomes larger, especially for the small sizes. There also observes the EER become relatively stable when the user size is larger than a certain number of users, and only small fluctuations with the error range are apparent. These results present that how the space of user profiles fills in as the number of users increases, and also demonstrate the influence of user space on sensor-based smartphone authentication may be minimal when the user space exceeds a certain size.

We explored the proposed approach across different types of smartphone screen sizes. The authentication with a larger screen size exhibits better accuracy than those with smaller screen sizes. This may be due to the fact that input actions on a larger area have better uniqueness and robustness than the ones on smaller areas. Currently, the mainstream smartphones have screen size around 5 inches; while in our evaluation, the authentication on the smartphones with screen sizes around 5 inches have a FAR of 5.01% and a FRR of 6.85%, which further presents a proof-of-concept analysis to demonstrate the effectiveness of this technique.

For authenticating a user’s identity through the motion-sensor behavior, one important thing is that the registration and authentication process should take place in a secure environment, to avoid impostors maliciously manipulating users’ profile data. In our system implementation, we consider a simple way to secure the profile data by storing it in an encrypted domain (using standard encryption techniques of AES), but this may lead to the risk of leaving the profile data exposed during each authentication. A securer way is to employ a transformation function to the profile data [44], and only store the transformed profile data. Besides, during the profile generating phase, users’ identity should be checked in some alternative way, and the production of sensor-behavior samples should be limited to a few days, not to weeks or months.

When behavior-based techniques are utilized for user authentication, it may raise concerns about user’s privacy. At least users should be aware that they are under observation, and should also understand that every security policy must imply a limitation of their privacy in some way [45]. Compared with other behavior-based authentication methods (e.g., keystroke dynamics may record users’ passwords and some sensitive textual information), the motion-sensor behavior analysis records the information of a smartphone’s posture and gesture change when the user inputs passcode on smartphone, and will have to be blurred for the privacy concern. The recorded sensor data would be stored in terms of sensor features and the corresponding timing information, and are made available exclusively to authentication process, which giving away little information about users’ smartphone activities and credentials.

There is still much space to improve the performance of sensor-based smartphone authentication. One way may be to analyze variability and noise in motion-sensor behavior, and then to develop effective methods to mitigate the impact of these factors, which are also critical to extract stable and discriminative behavior features. Other ways may be to clean the raw senor data of extraneous noise for obtaining high quality data, or to establish sophisticated pattern classifiers (e.g., the ensemble approach) that are robust to variable behavior data.

## Figures and Tables

**Table 1:** Sensors enabled in some popular smartphones.

Sensor	Samsung S6	iPhone 6	Nexus 7	Huawei P8
Gravity	√	√	√	√
Accelerometer	√	√	√	√
Gyroscope	√	√	√	√
Magnetometer	√	√	√	√
Light	√	√	√	√
Proximity	√	√	×	√
Fingerprint	√	√	×	×
Heart-Rate	√	×	×	×
Barometer	×	√	×	×
Compass	×	√	√	×
GPS	√	√	√	√

**Table 2:** Motion-sensor behavior features for each input action (with feature dimension in parentheses).

Category	Feature	Description
Descriptive Features	Mean	Mean value of overall time for a sensor-data sequence (1).
	Minimum	Minimum value of overall time for a sensor-data sequence (1).
	Maximum	Maximum value of overall time for a sensor-data sequence (1).
	Range	Range of overall time for a sensor-data sequence (1).
	Variance	Variance of overall time for a sensor-data sequence (1).
	Kurtosis	Width of peak for a sensor-data sequence (1).
	Skewness	Orientation of peak for a sensor-data sequence (1).
	Quantiles	Quantiles of a sensor-data sequence from 30% to 80% by step 10% (6).
	Cross-mean Rate	Degree of fluctuation for a sensor-data sequence (1).
Intensive Features	Energy	Intensity of a sensor-data sequence (1).
	Entropy	Dispersion of spectral distribution for a sensor-data sequence (1).

**Table 3:** Relative mutual information for sensor-behavior features.

No.	Sensor-Behavior Features	Mutual Information	No.	Sensor-Behavior Features	Mutual Information
1	Energy of accelerometer data in *y*-axis	0.8550	11	Max of accelerometer data in *x*-axis	0.8178
2	Entropy of accelerometer data in *y*-axis	0.8516	12	Variance of accelerometer data in *y*-axis	0.8119
3	Energy of accelerometer data in *z*-axis	0.8423	13	Range of accelerometer data in *x*-axis	0.8032
4	Entropy of accelerometer data in *z*-axis	0.8415	14	Range of accelerometer data in *y*-axis	0.8023
5	Energy of gyroscope data in *y*-axis	0.8403	15	Mean of gyroscope data in *x*-axis	0.7973
6	Energy of gyroscope data in *z*-axis	0.8337	16	Mean of gyroscope data in *y*-axis	0.7955
7	Entropy of gyroscope data in *y*-axis	0.8289	17	Min of gyroscope data in *x*-axis	0.7911
8	Entropy of gyroscope data in *z*-axis	0.8277	18	Max of gyroscope data in *x*-axis	0.7889
9	Mean of accelerometer data in *x*-axis	0.8196	19	Range of gyroscope data in *y*-axis	0.7888
10	Min of accelerometer data in *z*-axis	0.8187	20	Variance of gyroscope data in *z*-axis	0.7756

**Table 4:** FARs and FRRs in three different operational scenarios using three different classifiers (with standard deviation in parentheses).

Classifier	Hand-Hold-Input Scenario		Table-Hold-Input Scenario		Hand-Hold-Walk Scenario	
	FAR (%)	FRR (%)	FAR (%)	FRR (%)	FAR (%)	FRR (%)
One-Class SVM	5.01 (3.77)	6.85 (4.23)	7.85 (6.01)	9.27 (6.82)	10.95 (8.67)	13.12 (9.87)
Neural Network	7.79 (5.54)	9.15 (6.58)	10.83 (7.78)	11.87 (8.97)	13.42 (10.11)	15.13 (10.02)
k-Nearest Neighbor	10.13 (7.45)	12.25 (9.12)	13.11 (9.91)	16.43 (10.15)	18.23 (12.51)	21.32 (13.97)

**Table 5:** FARs and FRRs for Different Passcode Lengths (with standard deviation in parentheses).

Classifier	4-Digit Passcode		5-Digit Passcode		6-Digit Passcode	
	FAR (%)	FRR (%)	FAR (%)	FRR (%)	FAR (%)	FRR (%)
One-Class SVM	8.69 (6.21)	9.47 (6.92)	5.01 (3.77)	6.85 (4.23)	3.92 (2.03)	4.97 (2.87)
Authentication Time	1.52 s		2.89 s		3.31 s	

**Table 6:** FARs and FRRs at Different Training Data Size (with standard deviation in parentheses).

Training Data Size	FAR (%)	FRR (%)
10	13.78 (8.67)	16.13 (11.34)
20	9.02 (5.83)	10.33 (6.75)
30	6.97 (4.67)	8.79 (5.53)
50	5.01 (3.77)	6.85 (4.23)
70	4.13 (2.83)	5.27 (3.81)

**Table 7:** FARs and FRRs for Different Smartphone Screen Sizes (with standard deviation in parentheses).

Classifier	HongMi 1S (4.1 inches)		Samsung N7100 (5.5 inches)		Huawei Mate7 (6.1 inches)	
	FAR (%)	FRR (%)	FAR (%)	FRR (%)	FAR (%)	FRR (%)
One-Class SVM	9.74 (6.13)	11.09 (7.92)	5.01 (3.77)	6.85 (4.23)	4.53 (2.91)	5.89 (3.97)
